# Kidney, Prostate, and Bladder Cancer Burden Attributable to Tobacco Smoke Exposure in BRICS Countries from 1990 to 2021: A Systematic Analysis Based on the Global Burden of Disease Study

**DOI:** 10.3390/healthcare13233082

**Published:** 2025-11-26

**Authors:** Yushi Hou, Qian Zhang, Binglei Ma

**Affiliations:** 1 Department of Urology, Beijing Shijitan Hospital, Capital Medical University, Beijing 100038, China; houyushi@mail.ccmu.edu.cn (Y.H.); zq@bjsjth.cn (Q.Z.); 2 State Key Laboratory of Oncology in South China, Guangdong Provincial Clinical Research Center for Cancer, Sun Yat-sen University Cancer Center, Guangzhou 510060, China; 3 Department of Urology, Sun Yat-sen University Cancer Center, Guangzhou 510060, China

**Keywords:** tobacco smoke, genitourinary cancers, BRICS countries, Global Burden of Disease, cancer epidemiology

## Abstract

**Background:** While tobacco smoke remains a leading modifiable risk factor for urologic cancers, comprehensive assessments in BRICS countries are scarce. We aimed to quantify the burden of kidney, prostate, and bladder cancers attributable to tobacco exposure from 1990 to 2021 in BRICS countries. **Methods:** We estimated tobacco-attributable deaths, disability-adjusted life years (DALYs), years of life lost (YLLs), and years lived with disability (YLDs) for kidney, prostate, and bladder cancers in BRICS nations using data from the Global Burden of Disease Study 2021. The data were stratified by sex, age, and sociodemographic index (SDI) and analyzed for temporal trends using the estimated annual percentage change (EAPC). Forecasts until 2050 were produced using autoregressive integrated moving average (ARIMA) modeling. **Results:** In 2021, tobacco-related genitourinary cancers caused approximately 85,000 deaths and 1.8 million DALYs in BRICS countries. While age-standardized DALY rates declined in most countries, absolute burdens rose due to aging and population growth. Bladder cancer contributed the greatest burden, with notable sex disparities: males experienced significantly higher rates across all three cancers. Russia and South Africa had the highest age-standardized rates, while China and India bore the largest absolute burdens. YLLs dominated the total burden, but YLDs increased faster over time, indicating a growing need for survivorship care. **Conclusion:** Despite some progress in reducing age-standardized rates, tobacco-attributable urologic cancer burdens continue to rise in BRICS countries. Targeted tobacco control, early detection, and long-term survivorship care are essential to mitigate future impacts.

## 1. Introduction

Tobacco use remains one of the leading preventable causes of morbidity and mortality worldwide [1]. It significantly contributes to the global burden of non-communicable diseases, including cardiovascular diseases, chronic respiratory illnesses, and various forms of cancer [2]. Despite increasing awareness and global public health efforts, tobacco consumption remains widespread in many low- and middle-income countries, particularly among men [3].

Among tobacco-related cancers, kidney, prostate, and bladder cancers represent the most common malignancies of the genitourinary system [4]. These cancers are associated with substantial mortality, years of life lost, and rising treatment costs, especially in countries with aging populations and limited cancer screening capacity [5]. According to recent global cancer estimates, the incidence and mortality of these cancers have increased steadily over the past two decades, with wide disparities across regions and income levels [6].

There is strong epidemiological evidence linking tobacco smoke exposure to increased risk of both kidney and bladder cancers [7], with dose-dependent relationships observed in multiple meta-analyses. While the link between smoking and prostate cancer is less direct, studies have shown associations with more aggressive disease, higher mortality, and poorer treatment outcomes in smokers [8].

The Global Burden of Disease (GBD) study framework provides a comprehensive approach to quantifying disease burden and its associated risk factors across countries and time periods. Within this framework, the estimated annual percentage change (EAPC) is commonly used to describe temporal trends in age-standardized rates and allows for comparison of changing burdens among different populations.

The BRICS countries—Brazil, Russia, India, China, and South Africa—collectively account for over 40% of the global population and face a growing burden of non-communicable diseases [9]. Despite varying levels of tobacco control policy implementation, these countries share similar challenges, including high smoking prevalence (especially among men), demographic aging, limited access to early cancer detection, and underfunded cancer surveillance systems [10]. Few studies have systematically quantified the burden of kidney, prostate, and bladder cancers attributable to tobacco smoke exposure across BRICS countries using the GBD 2021 framework [11].

In this study, we aimed to estimate the deaths, disability-adjusted life years (DALYs), years of life lost (YLLs), and years lived with disability (YLDs) of kidney, prostate, and bladder cancers attributable to both active and secondhand tobacco smoke exposure in BRICS countries from 1990 to 2021, and to explore temporal trends, sex and age disparities, and national heterogeneity. These findings aim to inform regional policy responses and highlight priority areas for targeted tobacco control, early detection, and survivorship care within the BRICS context.

## 2. Methods

### 2.1. Data Sources

This study utilized data from the GBD 2021, which provides annual estimates for 371 diseases and 88 risk factors across 204 countries and territories from 1990 to 2021. All data were accessed via the Global Health Data Exchange (GHDx) results tool (https://ghdx.healthdata.org/gbd-results-tool [accessed on 19 February 2025]). The GBD estimates adhere to the Guidelines for Accurate and Transparent Health Estimates Reporting (GATHER) framework and are based on standardized methodologies for data collection, modeling, and risk attribution.

The GBD employs a comparative risk assessment framework using multiple data sources, including national surveys, cancer registries, vital registration systems, and household exposure assessments. These data are modeled using Bayesian meta-regression (DisMod-MR 2.1) to generate consistent estimates where empirical data are sparse.

### 2.2. Cancer Definitions

We assessed the burden of three genitourinary cancers based on the following International Classification of Diseases (ICD-10 and ICD-9) codes used in the GBD 2021:

Kidney cancer: C64–C65 (ICD-10), 189.0–189.1 (ICD-9);

Prostate cancer: C61 (ICD-10), 185 (ICD-9);

Bladder cancer: C67 (ICD-10), 188 (ICD-9).

The cancer burden metrics were estimated for each cancer site separately, including incidence, mortality, and DALYs, which are composed of YLLs and YLDs.

### 2.3. Exposure and Risk Attribution

Tobacco exposure included both active smoking and secondhand (passive) smoke exposure. Active smoking refers to current use of cigarettes, cigars, or other tobacco products, while secondhand smoke refers to inhalation of tobacco combustion products by nonsmokers in domestic or public environments.

Risk–outcome pairs were selected based on strong evidence of causality, following criteria from the World Cancer Research Fund (WCRF) and International Agency for Research on Cancer (IARC). Population exposure distributions were derived from nationally representative surveys and modeled using Bayesian meta-regression approaches in DisMod-MR 2.1.

The comparative risk assessment (CRA) framework was used to estimate the proportion of cancer burden attributable to tobacco smoke. This involved the computation of population attributable fractions (PAFs), which represent the proportion of disease burden that could be avoided if exposure to tobacco were reduced to the theoretical minimum risk exposure level (TMREL), defined as no smoking or exposure to secondhand smoke.

PAFs were calculated by combining the exposure distribution, relative risk (RR) functions from meta-analyses, and TMREL values. These fractions were applied to cancer-specific estimates of deaths, DALYs, YLLs, and YLDs to determine the burden attributable to tobacco smoke.

### 2.4. Indicators and Stratification

We reported the following key epidemiological indicators attributable to tobacco smoke exposure for each cancer type: deaths, DALYs, YLLs, and YLDs.

All estimates were age-standardized rates (ASRs) per 100,000 population using the GBD reference population, enabling cross-region and cross-year comparability. The results were stratified by sex, age group (in 5-year intervals from <20 to >95 years), and sociodemographic index (SDI) quintiles (low, low–middle, middle, high–middle, and high SDI).

All key estimates are presented with 95% uncertainty intervals (UIs), derived from 1000 posterior draws of the GBD model outputs.

### 2.5. Trend Analysis

We calculated the EAPC of ASRs using a log-linear regression model to evaluate long-term temporal changes in burden:

EAPC = 100 × (e^β^ − 1)

where β is the slope of the regression line fitted to the natural logarithm of the ASR over the calendar year. An EAPC > 0 with the lower bound of the 95% confidence interval (CI) > 0 indicates an increasing trend; an EAPC < 0 with the upper CI < 0 indicates a decreasing trend.

Confidence intervals (95% CIs) for all EAPC estimates were calculated from the regression model’s variance–covariance matrix, allowing interpretation of trend significance.

### 2.6. Uncertainty Estimation and Data Quality

All estimates were generated using 1000 posterior draws to calculate 95% uncertainty intervals (95% UIs), defined as the 2.5th and 97.5th percentiles of the posterior distribution.

The GBD applies multiple imputation and Bayesian-smoothing methods in countries with incomplete or poor-quality registry data (notably, India and South Africa) to reduce reporting bias. The remaining uncertainty due to underreporting was incorporated into the UIs.

### 2.7. Forecasting

Future projections were modeled using the autoregressive integrated moving average (ARIMA) method, which predicts future values of DALYs, YLLs, and YLDs based on historical temporal patterns from 1990 to 2021. The model performance was evaluated by comparing predicted versus observed values via 10-fold cross-validation.

### 2.8. Software

All data processing and statistical analyses were performed using R (version 4.2.2; R Foundation for Statistical Computing, Vienna, Austria) and Python (version 3.10; Python Software Foundation, Wilmington, DE, USA). Data visualization was conducted using ggplot2 (version 3.4.4; part of the tidyverse collection, RStudio, PBC, Boston, MA, USA), matplotlib (version 3.7.5; The Matplotlib Development Team, USA), and seaborn (version 0.12.2; The Seaborn Development Team, USA).

## 3. Results

### 3.1. Kidney Cancer Attributable to Tobacco Smoke

#### Burden and Temporal Trends

**Figure 1:**
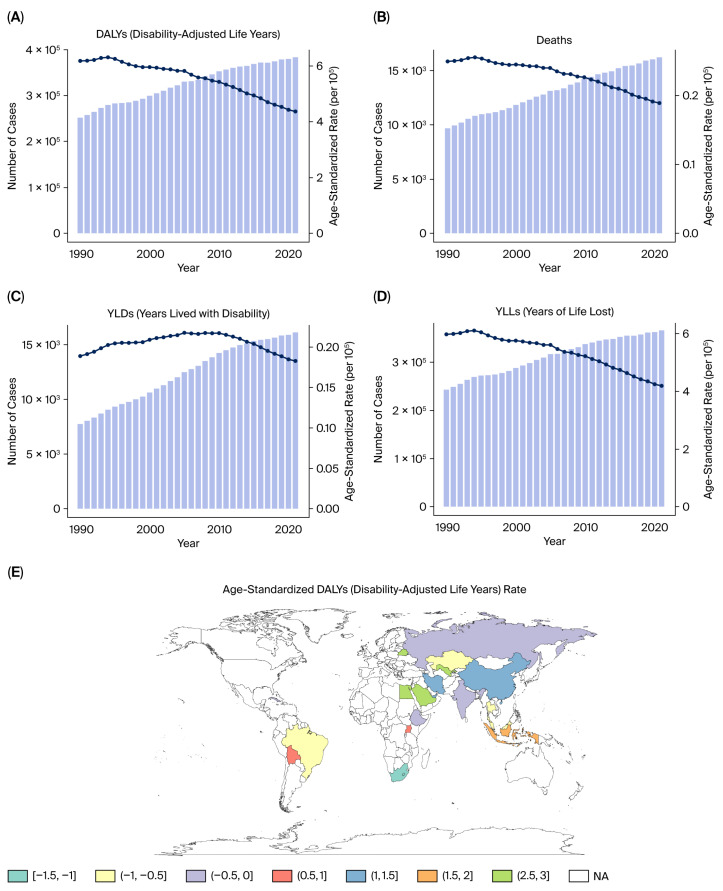
Kidney cancer burden attributable to tobacco smoke in BRICS countries (1990–2021). (**A**) Number of DALYs in BRICS countries. (**B**) Number of deaths in BRICS countries. (**C**) Number of YLLs in BRICS countries. (**D**) Number of YLDs in BRICS countries. (**E**) Proportional contribution of YLLs and YLDs to DALYs.

From 1990 to 2021, the burden in BRICS countries of kidney cancer attributable to tobacco smoke exposure exhibited a rising trend in both absolute numbers and age-standardized rates. In 2021, tobacco-related kidney cancer accounted for substantial health loss across BRICS countries, with an estimated over 15,000 deaths (95% UI: 13,000–17,000) and approximately 380,000 DALYs (95% UI: 340,000–410,000) (Figure 1A,B).

The burden of premature mortality (YLLs) accounted for the vast majority of DALYs across the study period. In 2021, YLLs attributable to tobacco-related kidney cancer in BRICS countries exceeded 300,000 (95% UI: 270,000–330,000), while YLDs remained much lower at approximately 15,000 (95% UI: 13,000–17,000) (Figure 1C,D). Despite this difference, YLDs showed a faster rate of increase over time, with EAPC values exceeding +4% in some countries, highlighting the growing importance of survivorship and disability management (Figure 1E).

Between 1990 and 2021, the age-standardized DALY rate of tobacco-related kidney cancer showed a marginal decline in high-income regions but remained stable or slightly increased in middle- and low-SDI countries. According to EAPC maps, most countries experienced an EAPC range between −1.5% and +3% for DALY rates, with a net increasing trend in low- and middle-income regions (Figure 1E).

### 3.2. Sex Differences

**Figure 2:**
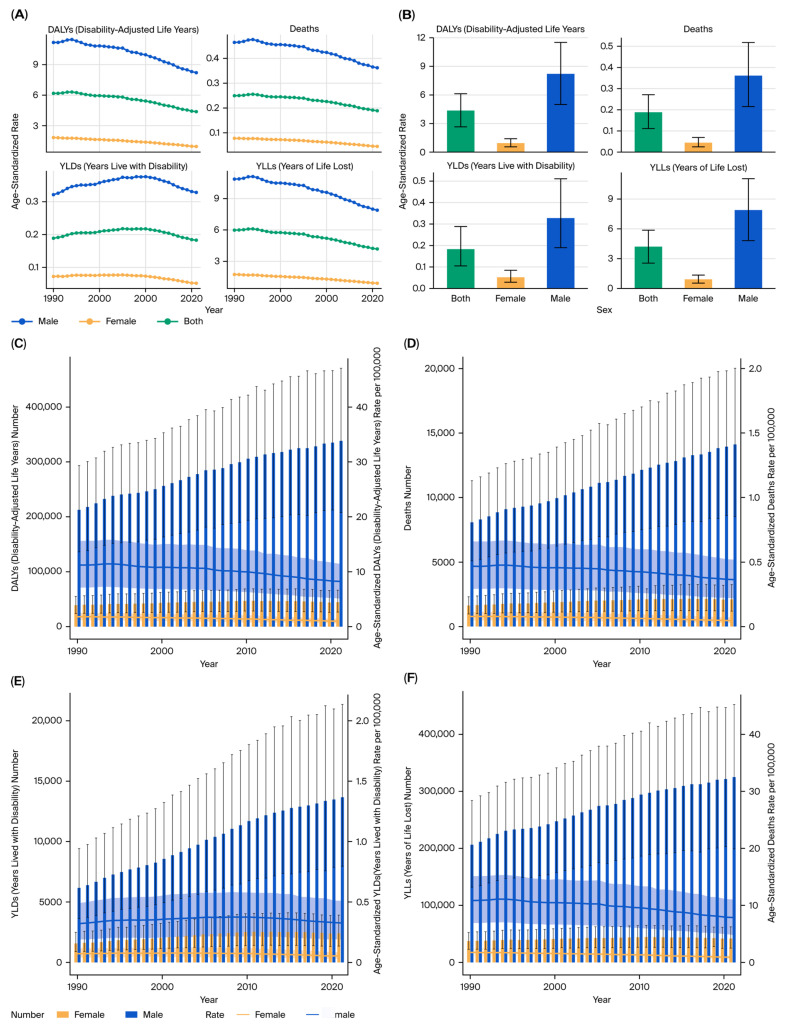
Sex-specific burden of kidney cancer attributable to tobacco smoke (2021). (**A**) Age-standardized DALY rate by sex. (**B**) Temporal trend of DALYs by sex. (**C**) Temporal trend of deaths by sex. (**D**) Temporal trend of YLLs by sex. (**E**) Temporal trend of YLDs by sex. (**F**) Ratio of male-to-female DALYs by year.

Sex-specific trends demonstrated a consistently higher burden in males than females across the study period. The male-to-female DALY ratio for kidney cancer remained at approximately 3:1 throughout the observation period (Figure 2A–F).

**Figure 3:**
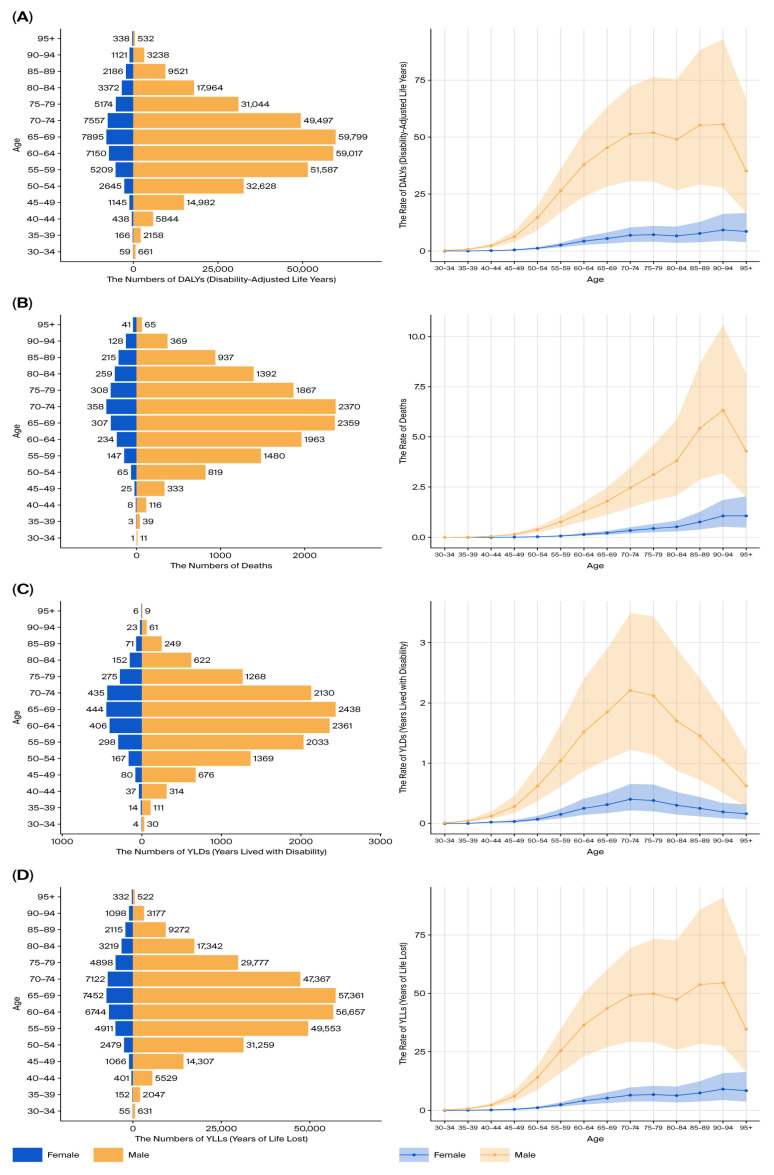
Age-specific burden of kidney cancer by sex (2021). (**A**) DALYs by sex and age group. (**B**) Deaths by sex and age group. (**C**) YLLs by sex and age group. (**D**) YLDs by sex and age group.

Additionally, age-specific analyses showed that male patients aged 60–74 years bore the greatest burden of tobacco-attributable kidney cancer DALYs, deaths, YLDs, and YLLs (Figure 3A–D). The burden among females peaked in a slightly older age group (65–79 years), but the magnitude remained lower than in males.

### 3.3. Age Distribution

**Figure 4:**
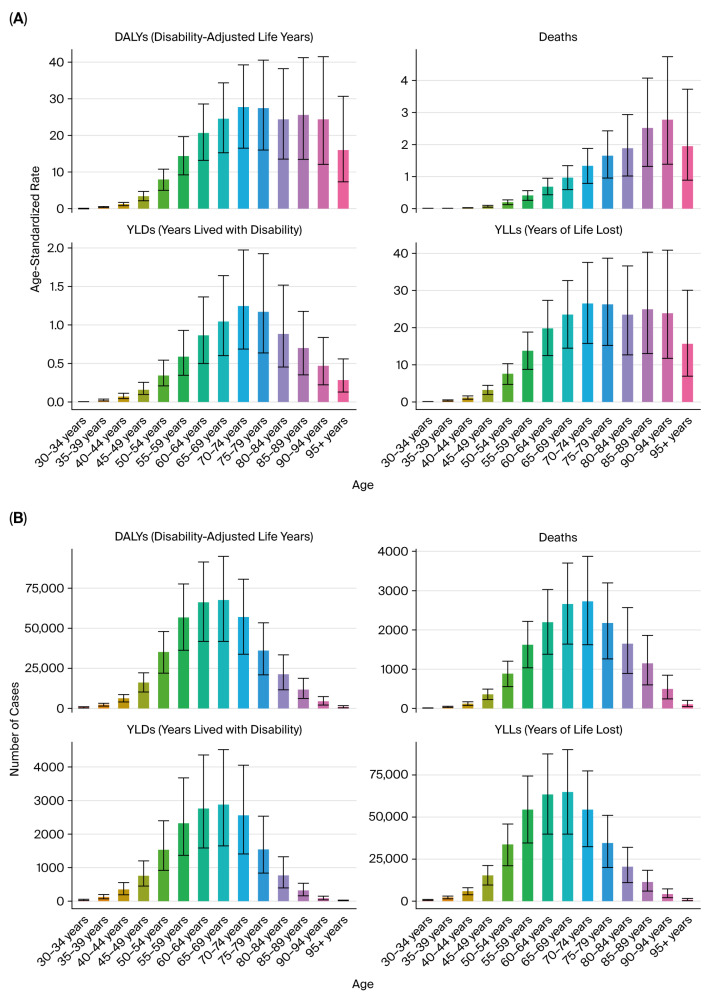
Age distribution of kidney cancer burden in 2021. (**A**) Age-specific DALYs. (**B**) Age-specific deaths.

The burden of kidney cancer increased markedly with age. In 2021, the number of DALYs and deaths attributable to tobacco exposure rose sharply after age 50, peaking in individuals aged 65–74 years (Figure 4A,B). Age-standardized DALY rates were minimal in individuals under 40, consistent with the latency period of tobacco-related carcinogenesis.

### 3.4. Geographic and SDI Patterns

**Figure 5:**
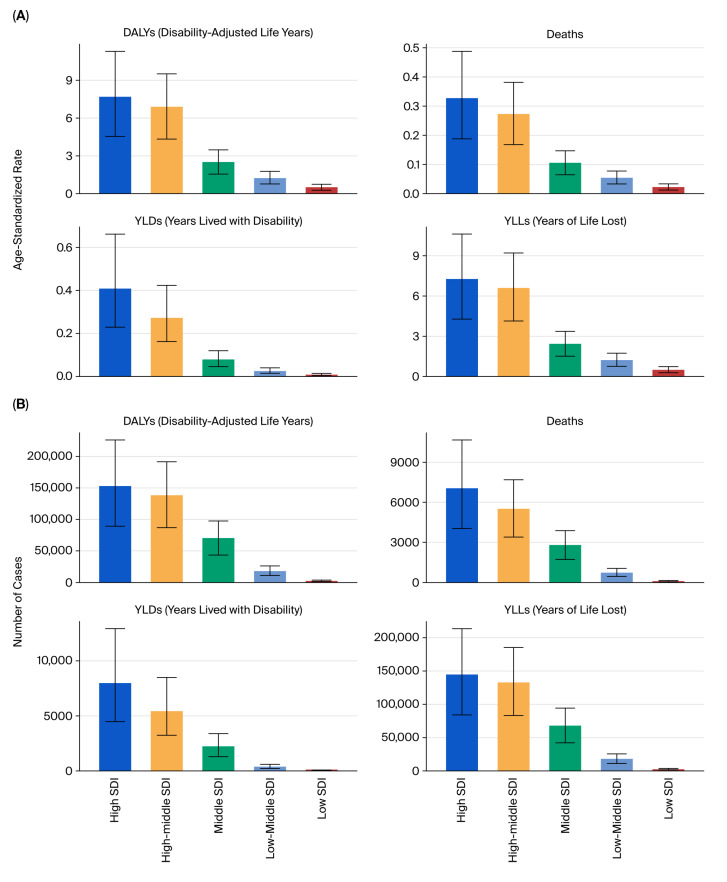

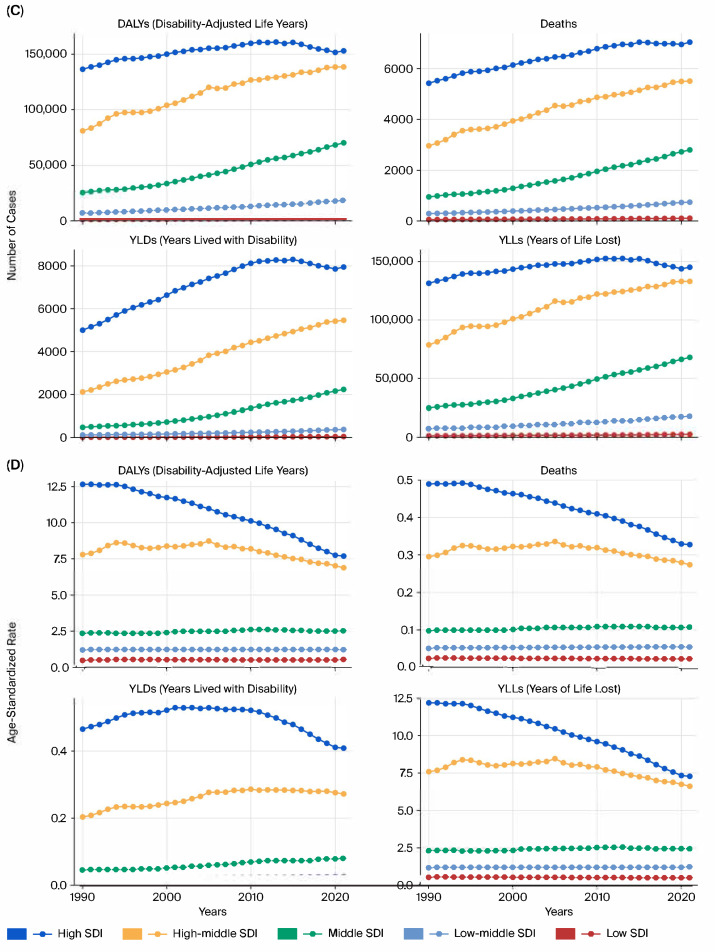
SDI-based burden of kidney cancer due to tobacco smoke (2021). (**A**) Total DALYs by SDI quintile. (**B**) Deaths by SDI quintile. (**C**) Age-standardized DALY rates by SDI. (**D**) Age-standardized death rates by SDI.

Geographically, high-SDI countries accounted for the largest number of total DALYs, deaths, YLLs, and YLDs in absolute terms, while middle- and low-middle SDI regions showed the most pronounced increases in age-standardized rates over time (Figure 5A–D).

**Figure 6:**
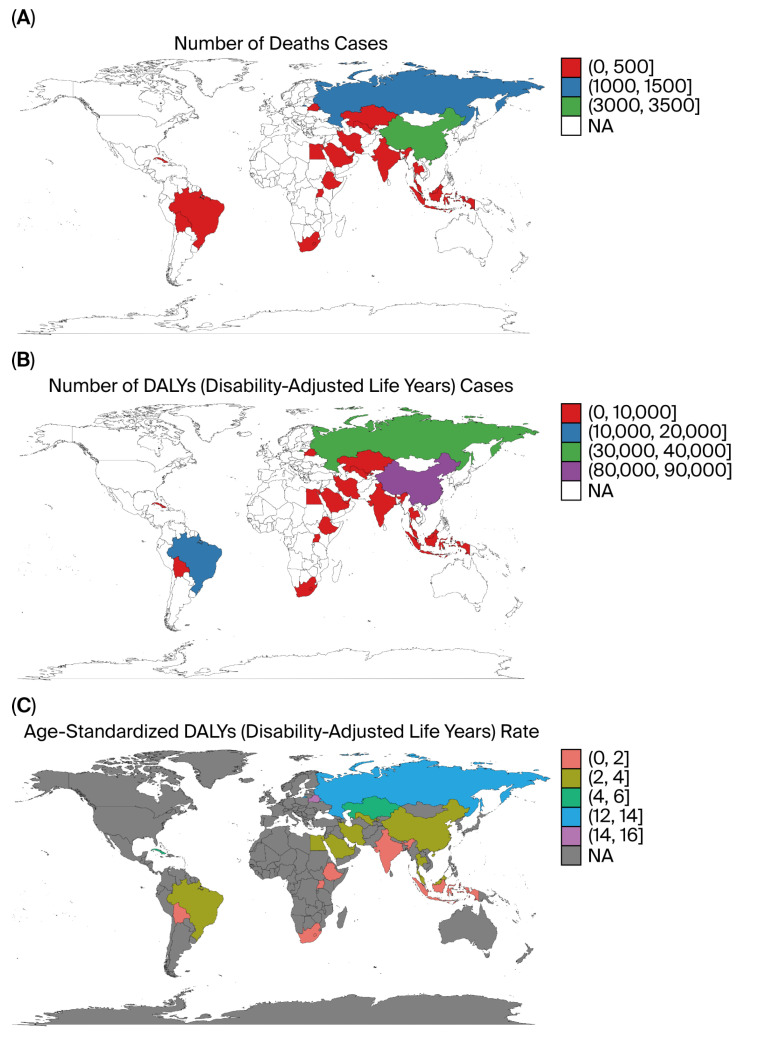
Top 20 countries with highest kidney cancer burden attributable to tobacco (2021). (**A**) Total DALYs. (**B**) Total deaths. (**C**) Age-standardized DALY rates.

At the national level, China, Russia, and Brazil ranked among the top contributors to DALYs and deaths attributable to tobacco-related kidney cancer (Figure 6A,B). However, the age-standardized rates were highest in Eastern Europe and Central Asia (Figure 6C), indicating a disproportionately higher per capita burden in those regions.

### 3.5. Estimated Future Burden

**Figure 7:**
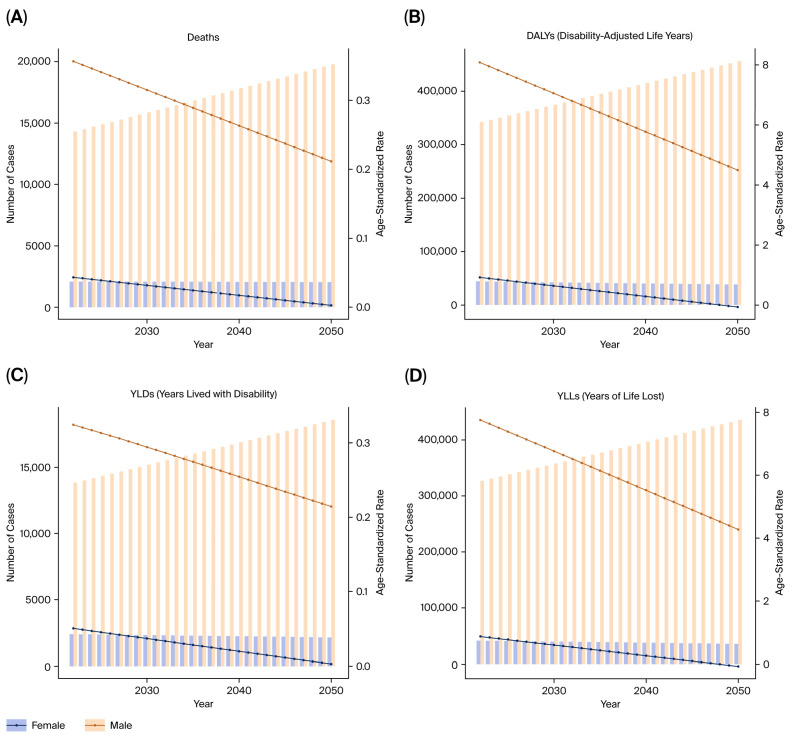
Forecasted burden of kidney cancer due to tobacco smoke (2021–2050). (**A**) Predicted DALYs. (**B**) Predicted deaths. (**C**) Predicted YLLs. (**D**) Predicted YLDs.

ARIMA-based forecasting models indicated a continuing upward trend in deaths, DALYs, YLLs, and YLDs from tobacco-attributable kidney cancer (Figure 7A–D).

## 4. Prostate Cancer Attributable to Tobacco Smoke

### 4.1. Burden and Temporal Trends

**Figure 8:**
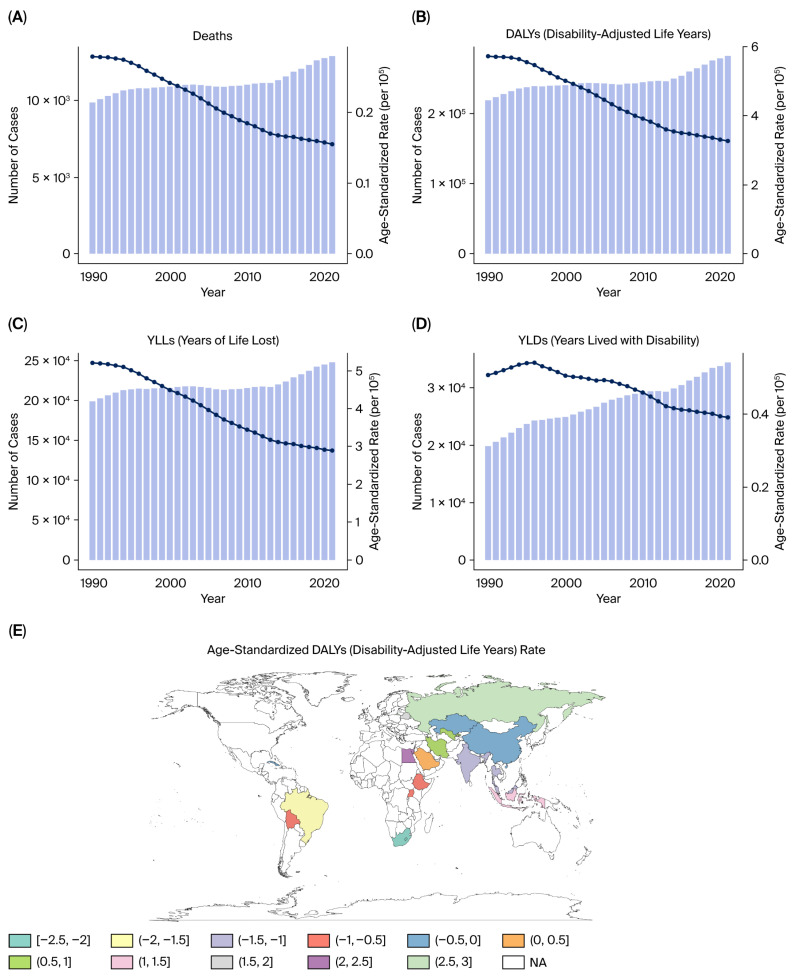
Temporal burden of prostate cancer attributable to tobacco smoke (1990–2021). (**A**) DALYs over time in BRICS countries. (**B**) Deaths over time in BRICS countries. (**C**) YLLs over time in BRICS countries. (**D**) YLDs over time in BRICS countries. (**E**) Proportional contribution of YLLs and YLDs to DALYs.

Between 1990 and 2021, prostate cancer attributable to tobacco smoke remained a persistent source of health loss in BRICS countries. In 2021, this cancer resulted in an estimated 10,000 deaths (95% UI: 8900–11,200) and 200,000 DALYs (95% UI: 180,000–220,000) (Figure 8A,B). YLLs accounted for the majority of the prostate cancer burden attributable to tobacco smoke, reaching 250,000 person-years (95% UI: 220,000–280,000). However, YLDs also contributed substantially, reaching approximately 30,000 (95% UI: 26,000–34,000) person-years, particularly due to chronic urinary complications and long-term treatment effects among survivors. Over time, YLDs have shown a more pronounced increase than YLLs, reflecting improved survival but growing disability in prostate cancer patients (Figure 8C,D).

Although the absolute burden increased moderately over the past three decades, age-standardized DALY rates remained relatively stable in BRICS countries, with substantial regional variability in trends. The EAPC in age-standardized DALY rates ranged from –2.5% to +3% across countries, indicating both rising and falling trends depending on geographic and socioeconomic contexts (Figure 8E).

### 4.2. Age Distribution

**Figure 9:**
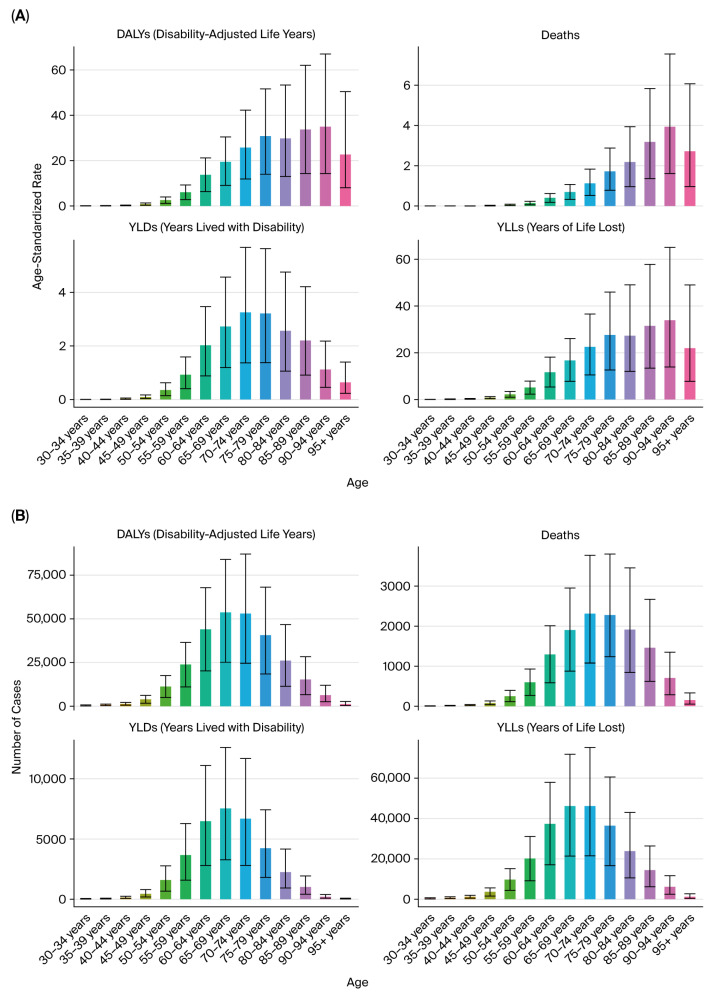
Age distribution of prostate cancer burden due to tobacco smoke (2021). (**A**) Age-specific DALYs. (**B**) Age-specific deaths.

The burden of prostate cancer attributable to tobacco smoke was predominantly concentrated in older adult populations, with a negligible impact in individuals under 50 years of age. In 2021, DALY numbers and age-standardized rates increased markedly in men aged 60 years and older, peaking in the 70–84 age range, consistent with prostate cancer’s natural history and tobacco-related cumulative risk (Figure 9A,B).

### 4.3. Geographic and SDI Patterns

**Figure 10:**
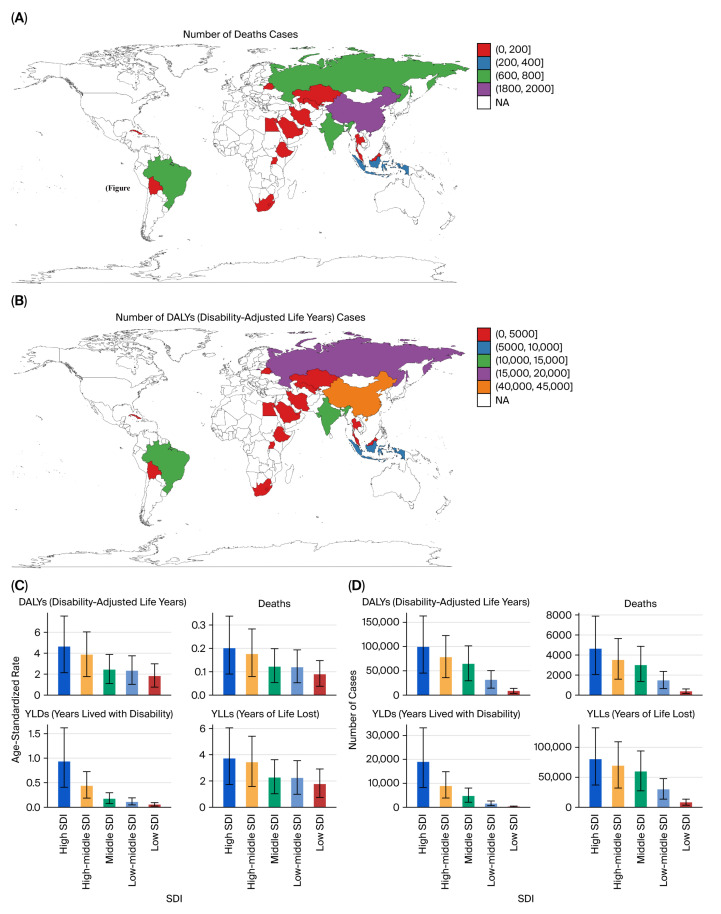
Geographic distribution of prostate cancer burden (2021). (**A**) Deaths by country. (**B**) Total DALYs by country. (**C**) Age-standardized DALY rate by SDI. (**D**) Total DALYs by SDI quintile.

In terms of absolute burden, high- and high–middle-SDI countries, China, Russia, India, and Brazil, accounted for the greatest number of DALYs and deaths due to prostate cancer linked to tobacco smoke (Figure 10A,B). However, some low- and middle-SDI countries showed increasing ASRs, indicating a shifting burden toward developing regions, in parallel with rising smoking prevalence and healthcare access disparities (Figure 10C,D).

### 4.4. Estimated Future Burden

Although tobacco-attributable prostate cancer remains relatively stable in many high-SDI settings, forecasting models suggest a possible increase in overall DALY numbers in the next decade in the absence of enhanced tobacco control efforts in BRICS countries.

**Figure 11:**
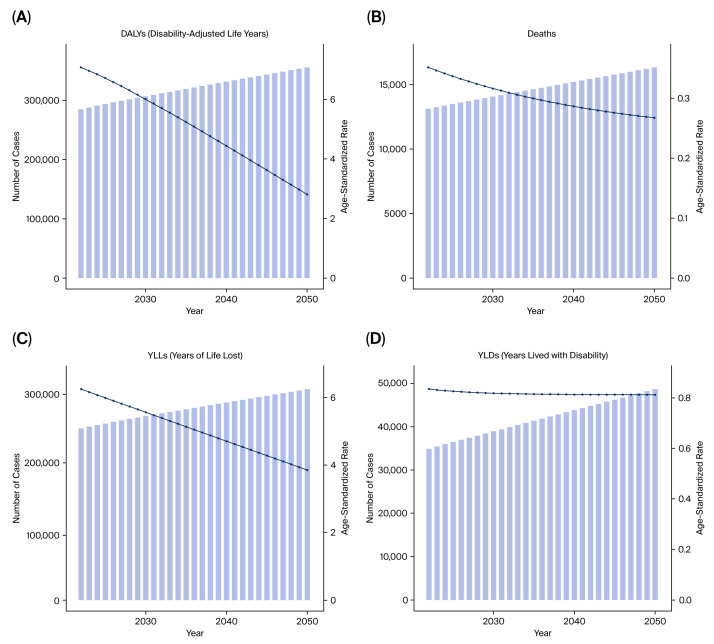
Projected burden of prostate cancer from tobacco exposure until 2050. (**A**) Predicted DALYs. (**B**) Predicted deaths. (**C**) Predicted YLLs. (**D**) Predicted YLDs.

Importantly, future trends in the burden structure are expected to shift. While YLLs may stabilize or decline modestly due to earlier detection and improved survival, YLDs are projected to rise steadily, reflecting a growing population of prostate cancer survivors living with chronic morbidity (Figure 11A–D).

## 5. Bladder Cancer Attributable to Tobacco Smoke

### 5.1. Burden and Temporal Trends

**Figure 12:**
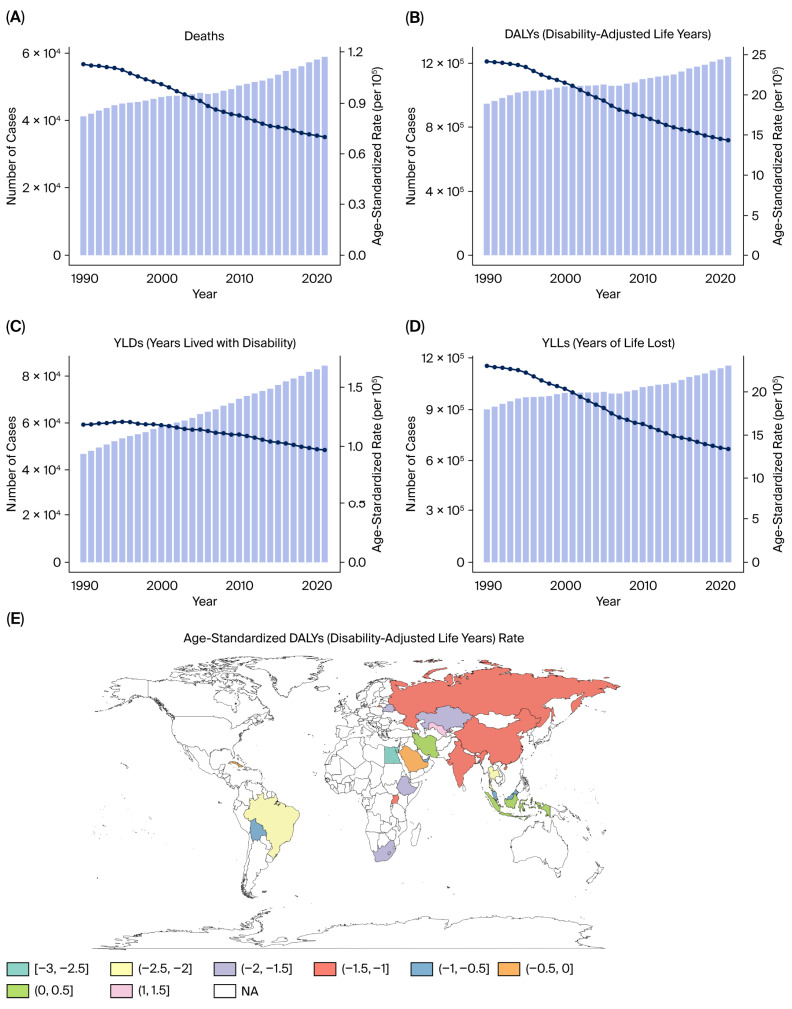
Temporal burden of bladder cancer due to tobacco smoke (1990–2021). (**A**) DALYs in BRICS countries. (**B**) Deaths in BRICS countries. (**C**) YLLs in BRICS countries. (**D**) YLDs in BRICS countries. (**E**) Proportion of YLLs vs. YLDs.

In 2021, bladder cancer attributable to tobacco smoke remained a major contributor to the cancer burden in BRICS countries. That year, it accounted for an estimated 60,000 deaths (95% UI: 54,000–66,000) and approximately 1.2 million DALYs (95% UI: 1.10–1.30 million) (Figure 12A,B).

Between 1990 and 2021, the absolute burden increased modestly; however, age-standardized DALY rates showed a declining trend in BRICS countries. The burden of bladder cancer was primarily driven by premature mortality. In 2021, YLLs exceeded 1.2 million person-years (95% UI: 1.10–1.30 million), far surpassing the disability component. However, YLDs also contributed approximately 80,000 person-years (95% UI: 70,000–90,000) (Figure 12C,D). The EAPC in age-standardized DALY rates ranged from –3.0% to 1.5% (Figure 12E).

### 5.2. Sex Differences

**Figure 13:**
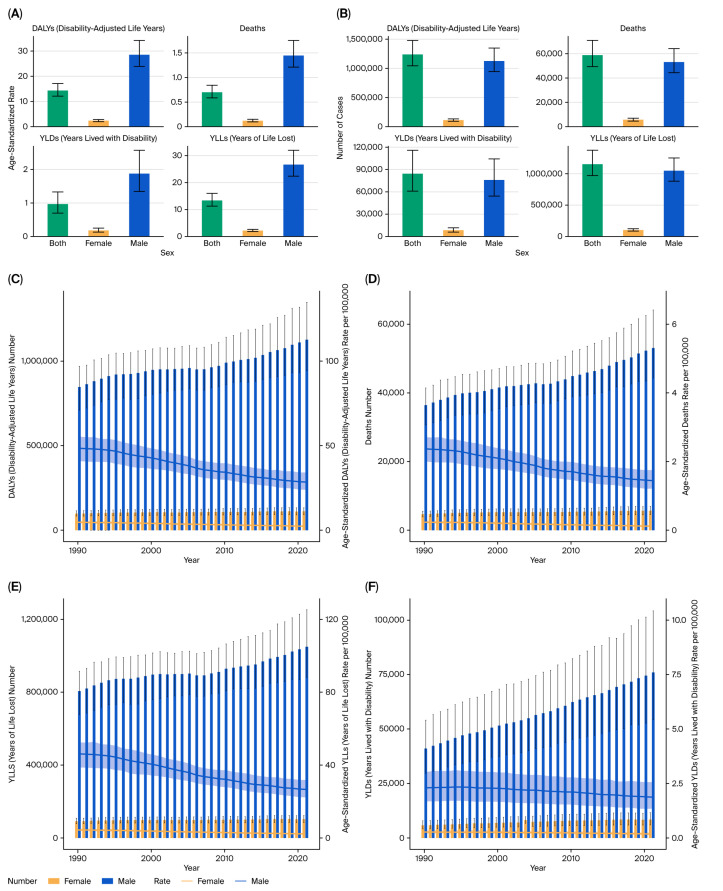
Sex-specific burden of bladder cancer due to tobacco smoke (2021). (**A**) Age-standardized DALY rate by sex. (**B**) Temporal DALY trend by sex. (**C**) Temporal deaths trend by sex. (**D**) Temporal YLLs by sex. (**E**) Temporal YLDs by sex. (**F**) Male-to-female burden ratio over time.

Sex-specific trends revealed a consistently higher burden in males than in females across all years from 1990 to 2021. In 2021, the age-standardized DALY rate for tobacco-attributable bladder cancer was substantially higher in men, reflecting both higher smoking prevalence and cumulative tobacco exposure (Figure 13A,B). This trend was consistent across all metrics, including DALYs, deaths, YLLs, and YLDs (Figure 13C–F).

**Figure 14:**
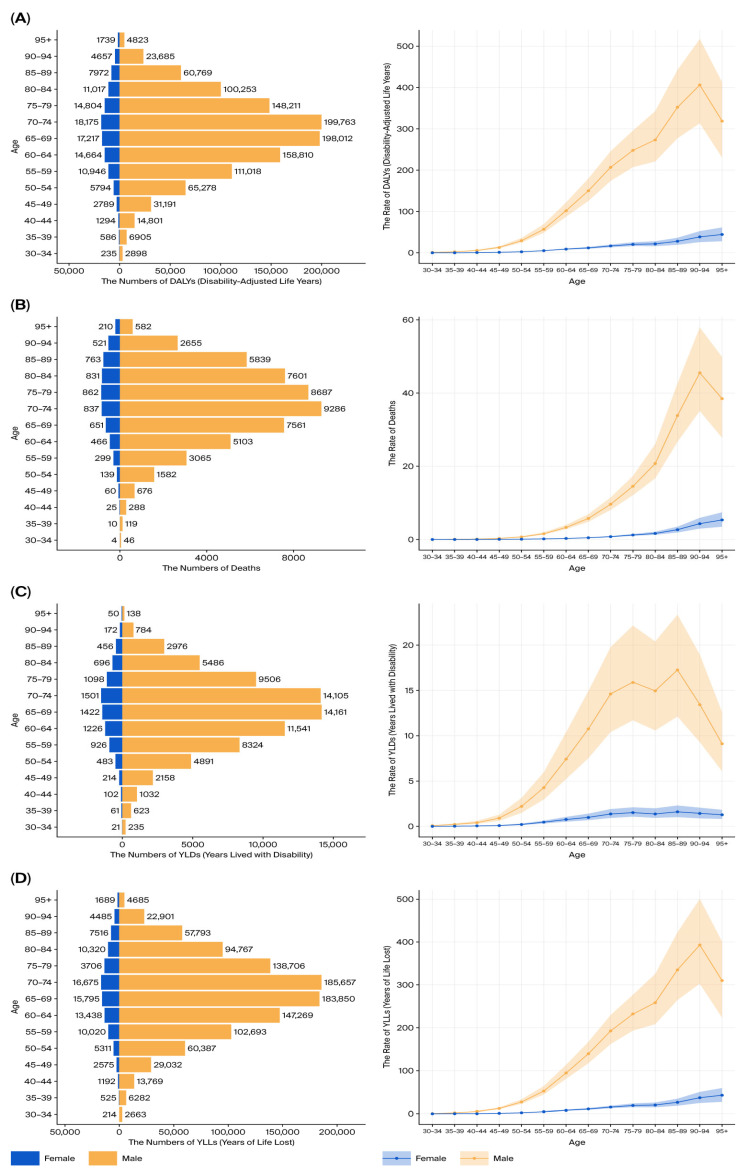
Age-specific bladder cancer burden by sex (2021). (**A**) DALYs by sex and age. (**B**) Deaths by sex and age. (**C**) YLLs by sex and age. (**D**) YLDs by sex and age.

Age-specific DALY rates demonstrated a sharp rise in males after age 60, peaking between 65 and 79 years. In females, the burden rose more gradually and peaked at a slightly older age. This pattern was evident across all burden components, including DALYs, deaths, YLLs, and YLDs (Figure 14A–D). Across all age brackets, the burden remained significantly higher in men.

### 5.3. Age Distribution

**Figure 15:**
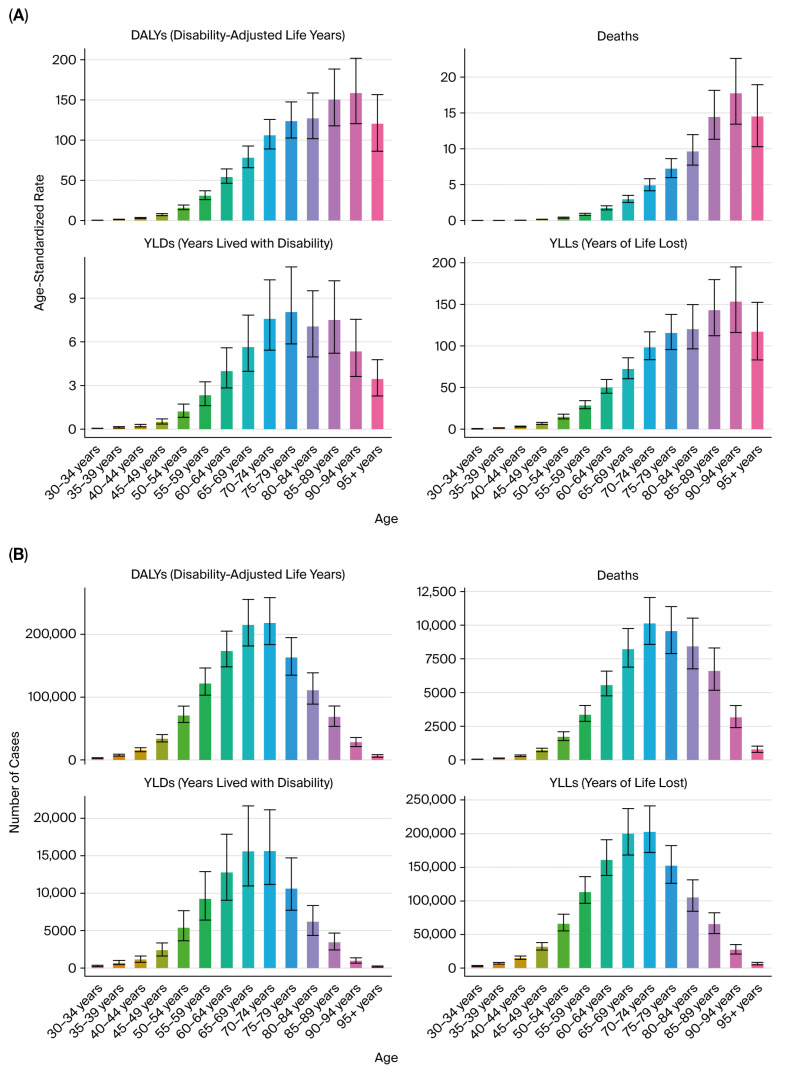
Age distribution of bladder cancer burden in 2021. (**A**) Age-specific DALYs. (**B**) Age-specific deaths.

As with other urologic cancers, the bladder cancer burden was age-dependent, with the majority of DALYs and deaths occurring in individuals aged 60 years and above. The burden peaked in the 70–79 age group. Younger age groups (<50 years) contributed minimally to the overall burden (Figure 15A,B).

### 5.4. Geographic and SDI Patterns

**Figure 16:**
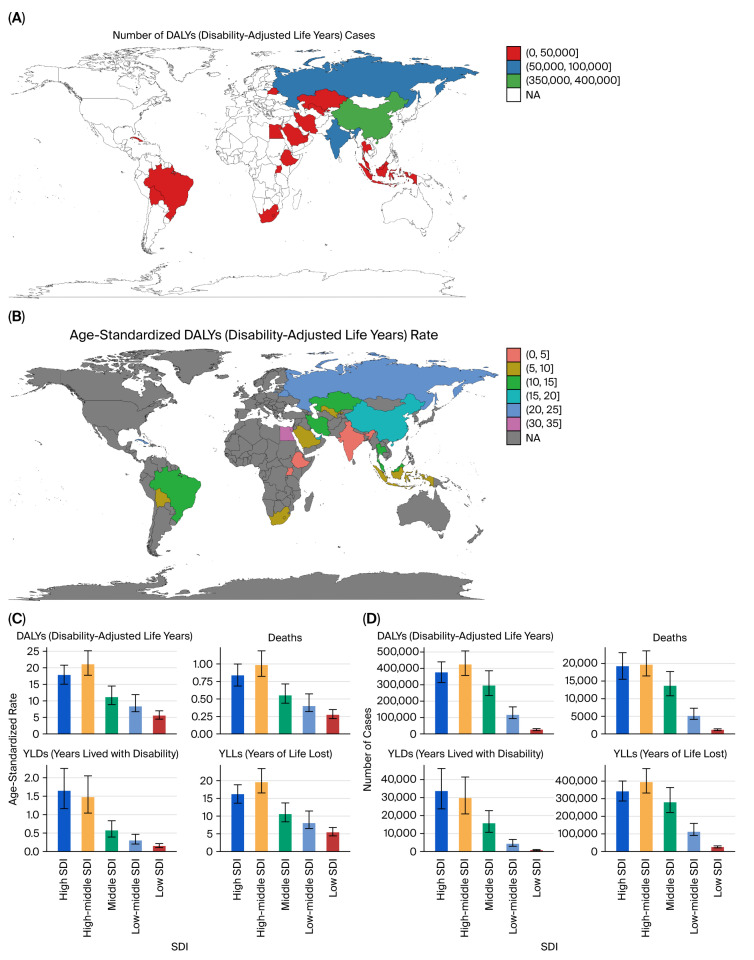
Geographic and SDI distribution of bladder cancer burden (2021). (**A**) DALYs by SDI. (**B**) Deaths by SDI. (**C**) Age-standardized DALY rate by SDI. (**D**) Age-standardized deaths by SDI.

In 2021, the highest absolute burden of tobacco-attributable bladder cancer occurred in high-SDI countries, particularly those with historically high smoking prevalence and well-developed cancer registries. Notable contributors included Russia, India, and China (Figure 16A,B). However, age-standardized DALY rates revealed that several middle-SDI countries—especially in Eastern Europe and Central Asia—had the highest per capita burden (Figure 16C,D).

### 5.5. Estimated Future Burden

**Figure 17:**
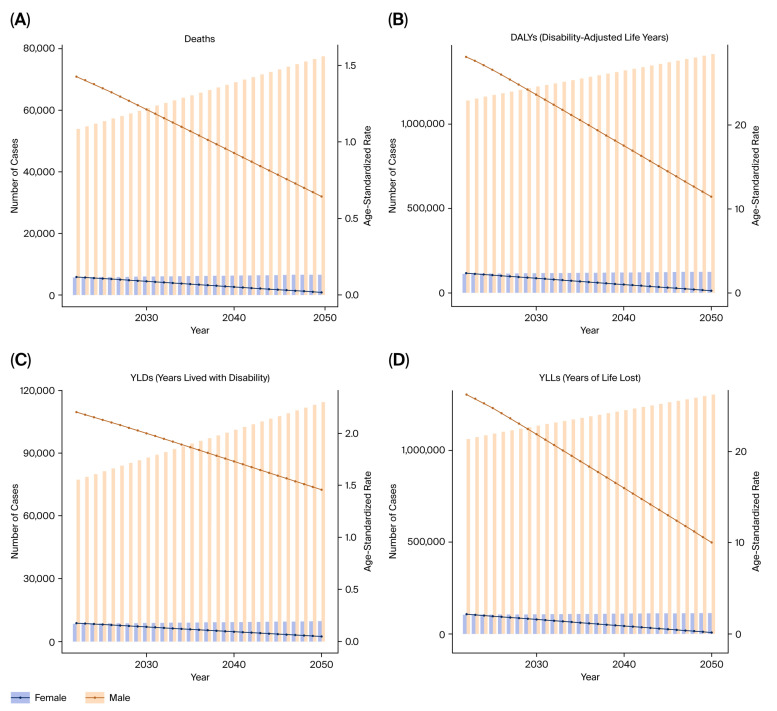
Forecasted burden of bladder cancer attributable to tobacco smoke until 2050. (**A**) Predicted DALYs. (**B**) Predicted deaths. (**C**) Predicted YLLs. (**D**) Predicted YLDs.

ARIMA-based forecasting models suggest that the overall burden of bladder cancer attributable to tobacco smoke may plateau or decline in many high-SDI countries. However, projections indicate a rising trend in YLDs, even as YLLs stabilize or fall, particularly in regions with improving cancer survival (Figure 17A–D).

## 6. Discussion

This study provides a comprehensive evaluation of the burden of kidney, prostate, and bladder cancers attributable to tobacco smoke exposure in the five BRICS countries from 1990 to 2021, based on data from the Global Burden of Disease 2021 framework. Across all three malignancies, tobacco exposure accounted for substantial proportions of deaths and DALYs, with persistently higher burdens in men and in high- or high–middle-SDI settings.

Despite improvements in public health infrastructure and the gradual decline in smoking prevalence in some regions, the cumulative burden of tobacco-related urological cancers remains high, reflecting both population aging and the delayed impact of smoking on cancer incidence.

Although certain countries have implemented national tobacco-control strategies, the overall tobacco-related cancer burden in BRICS remains considerable, emphasizing the need for integrated cancer-prevention and cessation efforts.

### 6.1. Comparative Patterns Across BRICS Countries

Marked heterogeneity was observed across the BRICS nations. China and Russia contributed the largest absolute number of deaths and DALYs, reflecting their large populations and high historical smoking prevalence, whereas South Africa exhibited the highest per capita rates, particularly for bladder cancer. India showed the lowest age-standardized rates but steady increases in absolute burden, likely due to demographic growth, urbanization, and delayed tobacco-control implementation. Brazil demonstrated gradual improvement after the introduction of stringent FCTC-aligned policies in the 2000s [1,2,11,12]. These inter-country differences mirror diverse epidemiological transitions, tobacco market penetration, and healthcare capacities within the BRICS bloc.

Sex-specific differences were pronounced: male-to-female DALY ratios exceeded 3:1 for kidney and bladder cancers [4,5,6], consistent with higher smoking prevalence, occupational exposures, and androgen-driven susceptibility that may influence detoxification enzyme activity and DNA repair capacity [13,14,15,16,17].

### 6.2. Biological and Exposure Mechanisms

Tobacco smoke remains one of the most pervasive and biologically potent carcinogenic exposures worldwide [7,8]. It contains numerous mutagens—including aromatic amines, nitrosamines, and polycyclic aromatic hydrocarbons—that induce DNA adducts, promote oxidative stress, and alter immune and vascular homeostasis.

For bladder cancer, these metabolites are filtered by the kidneys and concentrated in the urine, leading to direct urothelial contact and a mutation spectrum characterized by G → T transversions, consistent with tobacco-specific carcinogen signatures [18,19].

In the kidneys, chronic exposure to tobacco toxins induces tubular cell injury, endothelial dysfunction, and persistent inflammatory signaling, all of which can accelerate tumor initiation and progression [20,21].

Although the causal association with prostate cancer is less direct, several epidemiological studies have linked active smoking to higher Gleason grades, biochemical recurrence, and prostate-cancer-specific mortality [22,23,24].

Secondhand smoke exposure contributes meaningfully to disease burden, particularly among women and children in households with a high smoking prevalence, yet remains underrepresented in current global surveillance frameworks.

### 6.3. Socioeconomic and Health System Implications

The social gradient of tobacco-attributable cancer burden underscores both behavioral and structural inequities across BRICS countries [9,11]. High- and high–middle-SDI countries such as China, Brazil, and Russia contributed the largest absolute number of DALYs and deaths, reflecting more complete cancer registry coverage, greater diagnostic capacity, and aging populations. In contrast, low-SDI countries such as India and South Africa exhibited faster growth in age-standardized DALY rates [11], suggesting delayed enforcement of tobacco control measures, limited early detection, and under-resourced oncology infrastructure.

When compared with developed countries (i.e., high-SDI or high-income regions), the BRICS nations generally exhibit slower declines or stable trends in tobacco-attributable genitourinary cancers. Developed regions such as Western Europe and North America achieved earlier and more comprehensive implementation of tobacco control policies [2,9]—including taxation, advertising bans, and smoke-free regulations—resulting in sustained reductions in smoking prevalence and tobacco-related cancer mortality. In contrast, BRICS countries continue to face rising absolute burdens driven by population aging, delayed policy enforcement, and limited access to early-detection programs. This divergence highlights the importance of policy maturity and long-term investment in preventive infrastructure for achieving meaningful reductions in tobacco-related cancer burden.

Moreover, the relative risk coefficients (RRs) applied in the GBD model are largely derived from high-income cohorts; region-specific exposure–response functions in BRICS populations should be validated to improve precision. This limitation may partly explain the residual uncertainty and potential underestimation of the true regional burden.

### 6.4. Tobacco Control and Prevention Policies

Tobacco control remains the most effective and cost-efficient strategy for reducing the burden of urological malignancies. All BRICS countries are parties to the WHO Framework Convention on Tobacco Control (FCTC), yet the scale and rigor of implementation differ markedly [12]. Brazil and Russia have achieved significant reductions in smoking prevalence via taxation, graphic warnings, and advertising restrictions, whereas China and India continue to face challenges in enforcement, particularly in rural areas and among low-income groups.

Integrating cessation counseling, pharmacotherapy, and routine screening for high-risk individuals into primary care could reduce incidence while improving early-stage diagnosis.

Additionally, policies addressing secondhand smoke exposure—such as smoke-free public spaces, family education, and workplace bans—should be expanded to protect non-smokers, especially women and children.

Despite existing commitments, the growing burden of tobacco-related cancers suggests that FCTC enforcement and cross-sector coordination remain insufficient.

### 6.5. Interpretation and Public Health Implications

The three malignancies analyzed in this study—kidney, prostate, and bladder cancers—illustrate distinct yet converging patterns of tobacco-attributable burden. Although the age-standardized rates of bladder cancer have declined in several BRICS countries, the absolute number of DALYs continues to rise due to population aging and improved survival. Kidney and prostate cancers exhibit stable or increasing trends, suggesting an epidemiological shift toward older adults and chronic disease coexistence.

These observations indicate that primary prevention via tobacco control remains essential even in the context of improved treatment access. Sustained taxation, plain packaging, and nationwide smoke-free laws could collectively prevent hundreds of thousands of DALYs annually across BRICS. From a health system perspective, integrating tobacco cessation services into universal health coverage and non-communicable disease programs would yield high cost-effectiveness and improve long-term survivorship outcomes [2,9,11].

Moreover, the inclusion of secondhand smoke exposure metrics in routine cancer surveillance would allow more comprehensive risk monitoring, particularly for women and younger populations who are currently underrepresented in burden assessments.

### 6.6. Limitations

This analysis was subject to several methodological and data-related limitations inherent to the GBD approach.

First, relative risk coefficients used for estimating the burden of smoking-attributable cancers are predominantly derived from high-income countries and may not fully capture regional heterogeneity in exposure intensity, product type, or concurrent environmental risk factors.

Although the GBD framework applies 1000 posterior draws to quantify uncertainty, these estimates still rely on model assumptions that may underestimate the true variability in low- and middle-income settings.

Second, under-registration of cancer cases and incomplete death-certificate data, especially in India and South Africa, may contribute to residual underestimation despite the use of Bayesian meta-regression (DisMod-MR 2.1) and multiple imputation.

Third, the contribution of secondhand smoke could not be precisely quantified due to limited exposure data and potential misclassification.

Fourth, this study focused on aggregated national data and did not incorporate individual-level modifiers, such as occupation, alcohol consumption, genetic susceptibility, or healthcare accessibility.

Nevertheless, the application of standardized GBD methodology across countries and time provides valuable comparability for policy assessment and international benchmarking.

## 7. Conclusions

Tobacco-attributable kidney, prostate, and bladder cancers remain a substantial and persistent public health challenge in BRICS countries. Despite gradual declines in smoking prevalence in certain regions, the overall burden continues to increase due to demographic expansion, prolonged exposure histories, and incomplete enforcement of tobacco control policies. Strengthening FCTC implementation, expanding evidence-based cessation services, and promoting early detection programs targeting high-risk smokers are key strategies to mitigate future cancer burden. Policymakers should integrate tobacco-related cancer prevention into national NCD strategies, ensuring adequate funding and cross-sector collaboration. Future research should refine exposure–response relationships using region-specific cohorts and explore molecular mechanisms linking tobacco toxicity with renal and urothelial carcinogenesis, thereby supporting precision prevention and therapeutic innovation.

In summary, this study underscored the critical intersection between behavioral risk factors, aging populations, and health system responses by quantifying the long-term trends of three major urological cancers attributable to tobacco smoke in BRICS countries. Strengthened policy implementation and continuous surveillance will be essential to reverse these trends and achieve equitable cancer control across the BRICS region.

## Data Availability

The data presented in this study are openly available in the Global Burden of Disease (GBD) 2021 results tool, provided by the Institute for Health Metrics and Evaluation (IHME) at https://vizhub.healthdata.org/gbd-results (accessed on 19 February 2025).
